# Qualitative Analysis of Drug-Containing Plasma and its Application to Quantitative Analysis and Pharmacokinetic Study of Zexie Decoction Using UPLC-MS/MS

**DOI:** 10.3389/fchem.2022.815886

**Published:** 2022-02-22

**Authors:** Jiashuo Wu, Shunliang Zheng, Fangqing Zhang, Haonan Ruan, Haotian Xue, Jingxun Wang, Zhuangzhuang Li, Weiyi Jin, Weihua Wang, Jing Xia, Yue Shi

**Affiliations:** ^1^ Institute of Medicinal Plant Development, Chinese Academy of Medical Sciences and Peking Union Medical College, Beijing, China; ^2^ Mudanjiang Youbo Pharmceutical Co., Ltd., Mudanjiang, China; ^3^ College of Public Health, Hebei University, Baoding, China; ^4^ College of Public Health, Hebei Medical University, Shijiazhuang, China; ^5^ School of Pharmaceutical Sciences, Tsinghua University, Beijing, China; ^6^ Department of Pharmacognosy, School of Pharmacy, China Medical University, Shenyang, China

**Keywords:** qualitative analysis, quantitative analysis, pharmacokinetic study, zexie decoction, alisol

## Abstract

ZeXie Decoction (ZXD) is one of the traditional Chinese medicine formulas (TCMFs) comprising *Alisma orientalis* (Sam.) Juzep. (ZX) and *Atractylodes macrocephala* Koidz. (BZ) in 5:2 ratios and is widely employed in clinical applications since ancient times. In this study, UHPLC-QE-Orbitrap-MS was used for qualitative analysis of ZXD in rats’ plasma after a single oral dose of 750 mg/kg body weight. Afterward, UHPLC-Q-TRAP-MS/MS was used for simultaneous analysis of three bioactive chemical compounds including alisol A, alisol B, and alisol A 24-acetate in ZXD’s ethanol extract. Subsequently, the pharmacokinetic profiles of the three analytes were investigated in rat plasma utilizing UHPLC-Q-TRAP-MS/MS. The multiple reaction monitoring (MRM) mode for the three analytes were at m/z 508.4→383.2 for alisol A, m/z 490.4→365.2 for alisol B, and m/z 550.4→515.5 for alisol A 24-acetate. The analysis method was validated in terms of its accuracy, stability, repeatability, linearity, spiked recovery and matrix effect. As a result, twenty-five chemical constituents of ZXD were putatively identified in plasma, and rapid, sensitive, and accurate methods were established for the quantitative analysis and pharmacokinetic study of ZXD. The findings of this study can provide a scientific base for further study of *in vivo* pharmacokinetics of TCMFs.

## 1 Introduction

The traditional Chinese medicine formulas (TCMFs) have been employed clinically for a long time owing to their features of being “multi-component, multi-target, and multi-pathway” (Song et al., 2019). TCMFs, nowadays, are playing an important role in the diagnosis and treatment of a wide range of complicated disorders, including diabetes, non-alcoholic fatty liver disease, epilepsy, and osteoporosis (Ping Yang et al., 2020; Xu et al., 2020; Wu et al., 2021a; Xia et al., 2021). However, significant challenges are presented in terms of the quality control of TCMF due to the complex chemical constituents present in these TCMFs, thus limiting their wider clinical applicability. To understand the chemical constitution of TCMF in plasma, qualitative and quantitative analytical techniques, as well as pharmacokinetic studies, are routinely employed.

ZeXie Decoction (ZXD) is composed of *Alisma orientalis* (Sam.) Juzep. (Zexie, ZX) and *Atractylodes macrocephala* Koidz. (Baizhu, BZ) at a ratio of 5:2. It was recorded on “Synopsis of Golden Chamber”, a TCM masterpiece written by Zhongjing Zhang. Recent reports suggested that ZXD had a lipid-lowing and anti-inflammatory pharmacological effect, and it was frequently employed clinically to treat non-alcoholic fatty liver, atherosclerosis and hyperlipidemia clinically (Wu et al., 2021a). Although there are many investigations on the quali-quantitative analysis (Na Yang et al., 2020; Tang et al., 2021) and pharmacokinetic analysis (Tao et al., 2019) of the aqueous extract of ZXD and ZX, comparative investigations of their ethanol extracts are rather scarce.

In our previous study, a comparative study of the ethanol and aqueous extracts of ZXD based on UHPLC-Q-TOF-MS as well as an *in vitro* biological activity in the HepG_2_ cell line was performed. The results had suggested that the ethanolic extract of ZXD manifested a better hypolipidemic effect than the aqueous extract owing to the different chemical composition (Chang et al., 2021). Afterward, the ethanolic extract of ZXD was proved to have a therapeutic effect on non-alcoholic fatty liver disease in SD rats (Wu et al., 2021b). As a result, it was discovered that the ethanolic extract of ZXD had the significantly better potential for future investigation.

Herein, a total of twenty-five chemical constituents of ZXD were putatively identified in plasma using UHPLC-QE-Orbitrap-MS, three of which were selected for further quantitative analysis and pharmacokinetic study by using UHPLC-Q-TRAP-MS/MS. These three chemical constituents were putatively identified as alisol A, alisol B, and alisol A 24-acetate, respectively. Overall, rapid, sensitive, and accurate methods were established for the qualitative, and quantitative analysis and pharmacokinetic profile of ZXD.

## 2 Materials and Methods

### 2.1 Chemicals and Reagents

The dried rhizomes of ZX and BZ (Batch number: DD6081, DD8061) were supplied by Beijing Huamiao Pharmaceutical Co., Ltd. (Beijing, China). Three standards including alisol A, alisol B, alisol A 24-acetate (Batch number CHB180313, CHB180316, and CHB180315) were supplied by Chengdu Chroma-Biotechnology Co., Ltd. HPLC grade methanol and acetonitrile were purchased from Honeywell Burdick and Jackson. Ultra-pure water was obtained by using a super-pure water system (Beijing, China). Nimodipine was purchased from Macklin reagent Co., Ltd. (Shanghai, China).

### 2.2 Apparatus and Analytical Conditions

#### 2.2.1 Qualitative Analysis Using UHPLC-QE-Orbitrap-MS

Qualitative analysis was performed by using UHPLC equipped with the online degassing machine, quaternary gradient pump, column temperature chamber and automatic sampler. The heated electrospray ionization (HESI) source was used in conjunction with the Q Exactive PlusTM Orbitrap MS system (Thermo Scientific, Waltham, MA, United States). Separation of the analytes was carried out using Waters ACQUITY UPLC HSS T3 C*18* column (2.1 mm × 100 mm, 1.8 μm). The temperature of the analytical column was preset at 30°C. The injection volume was 5 μL and a flow rate of 0.2 ml/min was used throughout the experiment. Gradient elution was carried out with water with 0.1% (v/v) formic acid in water (solvent B) and acetonitrile (solvent A). The gradient elution was as follow: 0–10 min, 100% B; 10–20 min, 100–70% B; 20–25 min, 70–60%B; 25–30 min, 60–50% B; 30–40 min, 50–30%B; 40–45 min, 30–0% B; 45–60 min, 0 %B; 60–60.1 min, 0–100% B; 60.1–70 min, 100% B. The qualitative analysis was performed based on a positive ion mode or a negative ion mode at the range of m/z 100–1,500. The optimized parameters of MS were set as follows: aux gas heater temperature, 350°C; capillary temperature, 320°C; sheath gas flow, 40 arb; auxiliary gas flow rate, 15 arb; positive spray voltage, 3.2 kv; resolution of MS, 70,000; resolution of MS/MS, 17,500.

#### 2.2.2 Quantitative Analysis and Pharmacokinetic Study Using UPLC-Q-TRAP-MS/MS

The quantitative analysis and pharmacokinetic investigation were conducted using an AB SCIEX QTRAP 5500 triple quadrupole mass spectrometer (AB SCIEX, Foster, CA, United States). The separation of alisol A, alisol B, and alisol A 24-acetate was carried out using an ACQUITY HSS T3 column (2.1 × 100 mm, 2.5 μm) coupled to a I-Class UPLC system (Waters Corporation, Milford, MA, United States) equipped with a binary pump. The analytical column was set to 35°C. This experiment employed an injection volume of 10 μL and a flow rate of 0.2 ml/min. Gradient elution was carried out with 0.1% (v/v) formic acid in water (solvent B) and acetonitrile with 2 mM ammonium acetate (solvent A). The gradient elution was as follow: 0–6 min, 40–0% B; 6–8 min, 0–40% B; 8–12 min, 40% B. The following were the optimum MS parameters: The ESI ion source temperature was 500°C; the air curtain pressure was 30 psi; the collision activated dissociation (CAD) gas parameters were medium, and the ion spray voltage was 5500 V.

### 2.3 Preparation of Samples

#### 2.3.1 Extraction Method

The ZXD samples were prepared by combining ZX and BZ pieces in the (w/w) ratio of 5:2. After being crushed, pulverized, and decocted three times with a ten-times volume of 95% ethanol for 2 h each. After that, the decoctions were filtered, mixed, and concentrated under reduced pressure before being dried at 60°C for 48 h under a vacuum. The extraction rate of ZXD was 14.29% according to the following formula: Extraction rate = weight of ZXD powder/weight of ZX and BZ pieces.

#### 2.3.2 Pretreatment of Extract Samples

To prepare the working solutions of extract samples for quantitative analysis, 50 mg of ZXD ethanol extracts were diluted in 10 ml of 50% methanol to yield a solution with a concentration of 5 mg/ml. To obtain solutions with concentrations of 1 mg/ml, correctly weighted portions of the three standards were dissolved in 50% methanol to yield solutions with concentrations of 1 mg/ml. For measurement, the standard solutions were further diluted with acetonitrile.

#### 2.3.3 Pretreatment of Plasma Samples

The plasma samples were performed protein precipitation. 100 μL of the samples were spiked with 300 μL 20% methanol/60% acetonitrile/20% isopropanol/0.1% formic acid and vortexed for 1 min. Following that, the samples were centrifuged at 12,000 rpm for 10 min. After collection of the supernatant, it was spun dried and redissolved in 50% methanol for UPLC-MS analysis. The quality control (QC) samples of two levels were prepared in the same way at concentrations of 3 ng/ml and 400 ng/ml.

### 2.4 Methodology Validation

#### 2.4.1 Accuracy and Precision

Intra-day and inter-day precision were evaluated by two levels of QC samples on the same day (intra-day, *n* = 7) and three consecutive days (inter-day, *n* = 21).

#### 2.4.2 Stability

The short-term and long-term stability were evaluated by two levels of QC samples (*n* = 7) under the storage condition of 4°C for 24 h and −20°C for 14 days. The samples were further processed by freeze-thaw cycles repeated thrice.

#### 2.4.3 Specificity

To avoid endogenous interference, the specificity was determined by comparing representative multiple reaction monitoring (MRM) chromatograms of blank plasma to plasma spiked with alisol A, alisol B, alisol A 24-acetate, and an internal standard (IS).

#### 2.4.4 Linearity and the Threshold of Detection

Blank plasma samples containing alisol A, alisol B, alisol A 24-acetate and IS were prepared at eight different concentrations (2, 5, 10, 20, 50, 100, 200, 500 ng/ml for the three analytes). The linearity curves were plotted using the x-axis for concentrations and the y-axis for peak area. To obtain regression equations, least-squares linear regression was used. Furthermore, the limits of quantification and detection (LOQ and LOD) were determined, which was set to a signal-to-noise ≥10 and that ≥3.

#### 2.4.5 Spiked Recovery and Matrix Effect

Spiked recovery of spiked plasma samples was determined by comparing the observed concentrations of pre- and post-spiked plasma samples. Additionally, the matrix effect was calculated by comparing the observed concentrations of post-spiked plasma samples and IS adjusted solutions of pure standards.

### 2.5 Preparation of Experimental Animals

All the animal experiments were approved by the Institute of Medicinal Plant Development, Chinese Academy of Medical Sciences and Peking Union Medical College. A total of twelve Sprague-Dawley (SD) rats weighing 200 ± 20 g were provided by the Beijing Vital River Laboratory Animal Technology Co. Ltd. and were adaptively bred in an SPF-level environment for 3 days. The rats were randomly divided into two groups, including the treatment group (*n* = 6) and blank group (n = 6). Animals were fasted for 12 h before the experiment and were given free access to water. ZXD was dissolved in 0.5% sodium carboxymethylcellulose (CMC-Na) aqueous solution. Considering that the analytes in drug-containing plasma possess the characteristics of low content and strong interference, the maximum dosage was attempted (Zhou et al., 2010; Ying et al., 2020). The concentration of 150 mg/ml was adopted, which also showed flowability for administration. The volume of administration was 5 ml/kg, and thus ZXD was delivered orally to rats in the treatment group at a single dose of 750 mg/kg body weight. As a negative control, distilled water was given to the blank group rats. Animals were anesthetized with ether inhalation and blood samples were obtained in EDTA-anticoagulant tubes at the following time points: pre-dose, 20 min, 40 min, 1.5, 2, 3, 4.5, 6, 8, 10, 12, and 24 h. Plasma was collected from the blood samples by centrifugation at 3,000 g for 10 min and stored at −80°C for subsequent analysis. After the final time point of blood collection, rats were sacrificed via anesthetic overdose.

For the qualitative examination of plasma-containing drugs, a portion of plasma samples collected at various time points was combined. The remainders of the samples were used for the pharmacokinetic study to analyze the dynamic processes of chemical constituents in animals.

### 2.6 Data Analysis

The LC-MS data were examined using the Xcalibur workstation’s Qual Browser and Quan Browser (Thermo Scientific, United States, Version 4.1). SPSS 23.0 software (SPSS Inc., United States, Version 23) was used for statistical analysis, and sample concentrations were expressed as mean ± SD. The pharmacokinetic profile was depicted by GraphPad Prism software (Bethesda, United States, Version 6.02), and the pharmacokinetic parameters were calculated by Phoenix WinNonlin software (Certara, Corp., Version 6.3).

## 3 Results and Discussion

### 3.1 The Selection Criteria of Analytes

In a pre-experiment, we proved that alisol A, alisol B, and alisol A 24-acetate had significant lipid-lowing effect *in vitro* through an oleic acid-reduced lipid accumulation model in the HepG_2_ cell line (Supplementary Figures S7–S9). Moreover, the lipid-lowing and anti-inflammatory activities of alisol A, alisol B, and alisol A 24-acetate have been broadly reported recently (Zeng et al., 2016; Fei et al., 2018; Ho et al., 2019). Considering ZXD-mediated treatment of NAFLD and a number of other diseases related to dysregulation of lipid metabolism (Song et al., 2014; Xue et al., 2014; Wu et al., 2021b; Chang et al., 2021), alisol A, alisol B and alisol A 24-acetate were selected to analyzed in the present study.

### 3.2 Optimization of Analysis Conditions

**FIGURE 1:**
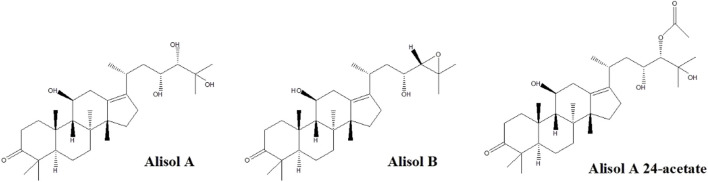
Chemical structures of alisol A, alisol B, and alisol A 24-acetate.

Figure 1 depicts the chemical structures of the three analytes. Different kinds of mobile phases were compared to screen the optimized conditions. The eluting solvent system consisting of water with 0.1% (v/v) formic acid in water (solvent B) and acetonitrile (solvent A) was found suitable to improve the separation of components. The recovery was found not satisfactory if we solely used acetonitrile or methanol for protein precipitation. Therefore, we selected 20% methanol/60% acetonitrile/20% isopropanol/0.1% formic acid mixture to attain better precipitation results.

For the qualitative analysis, the high-resolution mass spectra based on UHPLC-QE-Orbitrap-MS was used to screen the maximum chemical constituents in plasma. The gradient elution procedures with a total run time of 70 min were adopted for a better separation effect. Additionally, both positive and negative ion modes of operation were carried out. It was demonstrated that a greater number of peaks can be recorded in positive ion mode, thus was chosen for formal trials.

For the quantitative analysis and pharmacokinetic study, targeted quantitative mass spectra based on UPLC-Q-TRAP-MS/MS were employed for accurate quantification. The gradient elution procedures of 12 min were adopted to accommodate both efficiency and separation effect. Additionally, the collision energy and de-clustered voltage parameters of the best fit in MRM mode were investigated to assure the highest possible relative abundance of the ion components (Table 1).

**TABLE 1:** The mass spectrometry parameters for analytes.

t_R_ (min)	Analytes	Precursor ion (m/z) → product ion (m/z)	Collision energy (eV)	De-clustered voltage (V)
4.33	Alisol A	508.4 → 383.2	17	80
6.10	Alisol B	490.4 → 365.2	24	70
5.18	Alisol A 24-acetate	550.4 → 515.5	23	90
3.46	Nimodipine (IS)	419.0 → 343.3	13	110

**FIGURE 2:**
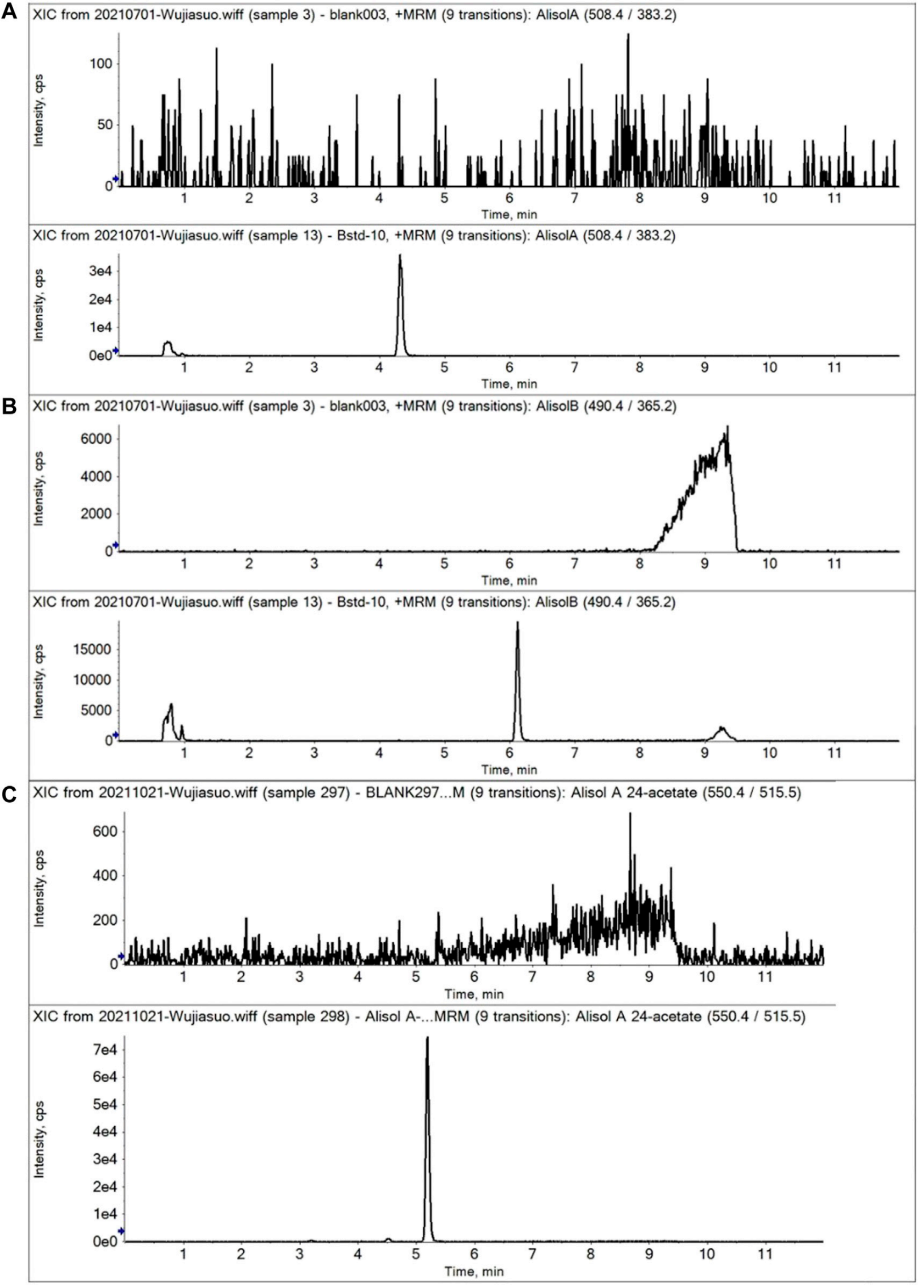
Representative MRM chromatograms of alisol A, alisol B, and alisol A 24-acetate in plasma samples. **(A)**. Alisol A; **(B)**. Alisol B; **(C)**. Alisol A 24-acetate. (Up-down, blank plasma and that spiked three analytes).

For the methodology validation, Table 2 shows the accuracy and precision of QC samples, indicating that this assay has an acceptable precision. Additionally, the samples were stable in the presence of long- and short-term storage, as well as three-freeze–thaw cycles (Table 3). At the retention times of alisol A (4.33 min), alisol B (6.10 min), alisol A 24-acetate (5.18 min), and IS (3.46 min), no endogenous interferences were identified (Figure 2). The linearity curves of the three analytes exhibited excellent linearity (R^2^ > 0.99) (Table 4). The LOD and LOQ met the requirements of the present quantitative analysis (Table 4; Supplementary Figures S1–S6). The spiked recovery and matrix effect were determined from 90–110% (Table 5).

**TABLE 2:** Accuracy and precision of QC samples.

Analytes	Nominal concentrations (ng/ml)	Intra-day (*n* = 7)			Inter-day (*n* = 21)		
		Observed concentration (ng/ml) (Mean ± SD)	Accuracy (%) (Mean ± SD)	RSD (%)	Observed concentration (ng/ml) (Mean ± SD)	Accuracy (%) (Mean ± SD)	RSD (%)
Alisol A	3	3.30 ± 0.25	94.10 ± 7.64	8.04	3.67 ± 0.35	104.80 ± 9.94	9.45
	400	432.71 ± 34.82	108.36 ± 8.78	8.05	434.48 ± 27.65	108.69 ± 6.91	6.36
Alisol B	3	3.15 ± 0.31	105.03 ± 10.39	10.00	3.22 ± 0.27	107.27 ± 8.95	8.38
	400	405.43 ± 35.77	101.40 ± 9.07	8.82	405.81 ± 37.06	101.42 ± 9.24	9.13
Alisol A 24-acetate	3	3.30 ± 0.25	110.39 ± 8.42	7.57	3.32 ± 0.19	110.60 ± 6.46	5.75
	400	427.57 ± 25.20	106.93 ± 6.38	5.89	423.48 ± 30.34	105.80 ± 7.53	7.16

**TABLE 3:** Stability of QC samples.

Analytes	Nominal concentrations (ng/ml)	Short term (*n* = 7)			Long term (*n* = 7)			Freeze-thaw (*n* = 7)		
		Observed concentration (ng/ml) (Mean ± SD)	Accuracy (%) (Mean ± SD)	RSD (%)	Observed concentration (ng/ml) (Mean ± SD)	Accuracy (%) (Mean ± SD)	RSD (%)	Observed concentration (ng/ml) (Mean ± SD)	Accuracy (%) (Mean ± SD)	RSD (%)
Alisol A	3	3.33 ± 0.11	111 ± 3.7	3.24	2.94 ± 0.20	97.75 ± 6.45	6.76	3.10 ± 0.31	103.37 ± 10.38	10.05
	400	442.14 ± 18.4	110.57 ± 4.31	4.16	405.50 ± 10.84	101.35 ± 2.64	2.76	400.00 ± 6.26	99.85 ± 1.46	1.57
Alisol B	3	3.16 ± 0.13	105.29 ± 4.39	4.17	2.65 ± 0.21	88.35 ± 6.95	8.01	3.09 ± 0.33	103.93 ± 10.96	9.65
	400	434.29 ± 14.21	108.71 ± 3.45	3.27	387.17 ± 38.24	96.68 ± 9.43	9.88	409.67 ± 30.31	102.55 ± 7.74	7.29
Alisol A 24-acetate	3	3.29 ± 0.15	109.71 ± 4.86	4.50	3.06 ± 0.24	101.88 ± 8.04	7.86	3.06 ± 0.21	102.02 ± 7.14	6.83
	400	444.57 ± 10.94	111.29 ± 2.5	2.46	430.00 ± 11.93	107.5 ± 3.02	2.77	413.17 ± 24.39	104.3833 ± 6.06	5.90

**TABLE 4:** Linearity of the three analytes.

Analytes	Regression equations	Correlation coefficient (R^2^)	Concentration range (ng/ml)	LOD (ng/ml)	LOQ (ng/ml)
Alisol A	*y* = 3.16 × 10^−4^ *x* + 1.50 × 10^–6^	0.994	2–500	1	2
Alisol B	*y* = 2.09 × 10^−4^ *x* + 3.76 × 10^–5^	0.997	2–500	1	2
Alisol A 24-acetate	*y* = 7.23 × 10^−4^ *x* + 6.04 × 10^–5^	0.995	2–500	0.5	2

**TABLE 5:** Spiked recovery and matrix effect of the three analytes.

Analytes	Spiked concentrations (ng/ml)	Spiked recovery (Mean ± SD, *n* = 5)	RSD (%)	Matrix effect (Mean ± SD, *n* = 5)	RSD (%)
Alisol A	3	0.9952 ± 0.0276	2.77	0.9559 ± 0.0671	7.02
—	400	0.9401 ± 0.0528	5.61	0.9153 ± 0.0796	8.70
Alisol B	3	0.9715 ± 0.0992	10.21	1.0452 ± 0.049	4.69
—	400	0.9655 ± 0.1352	14.00	0.9915 ± 0.0591	5.96
Alisol A 24-acetate	3	0.9324 ± 0.0805	8.64	0.9308 ± 0.0488	5.24
—	400	0.9591 ± 0.1124	11.72	1.0234 ± 0.0695	6.79

### 3.3 Results of Qualitative Analysis

The qualitative data were analyzed by Excalibur software. In the positive MS^n^ spectra, the error values of quasi-molecular ions, including (M + H)^+^and [M + Na] ^+^, were calculated and the values lying within 15 ppm were selected to analyze the fragment ions in the light of previous reports and chemical databases. Afterward, the probable chemical structure was determined. Twenty-five chemical constituents of ZXD were putatively identified in drug-containing plasma in total. The details such as retention time, putative identification, and molecular formula are given in Table 6.

**TABLE 6:** The putatively identified chemical constituents in drug-containing plasma by *UHPLC-QE-Orbitrap-MS*.

Peak number	t_R_ (min)	Putative identification	Molecular formula	Error (ppm)	MS^1^ [M + H]^+^/[M + Na]^+^	Fragment ions collected in positive mode	Origin	References
1	19.77	Atractylon	C_15_H_20_O	1.143	217.1589	199.1485, 189.1641, 175.1120, 133.1014, 107.0857	BZ	Zhang et al. (2018)
2	20.97	Atractylenolactam	C_15_H_19_NO	2.126	230.1544	159.0809	BZ	Chang et al. (2021)
3	24.21	Atractylenolide III	C_15_H_20_O_3_	−0.526	249.1484	231.1379, 203.1432, 189.1275, 175.1118, 161.0962, 147.0807	BZ	Chang et al. (2021)
4	25.68	Atractylenolide II	C_15_H_20_O_2_	−1.479	233.1538	215.1431, 205.1590, 189.0913, 187.1485, 177.1277, 159.1171, 145.1013	BZ	Liu et al. (2010)
5	30.91	Alisol C	C_30_H_46_O_5_	0.186	487.3434	469.3324, 451.3225, 397.2735, 379.2648	ZX	Liu et al. (2010)
6	31.87	Atractylenolide VI	C_15_H_22_	0.752	203.1796	161.1328, 147.1169, 133.1013, 119.0856	BZ	Chang et al. (2021)
7	32.87	11-deoxy-alisol C	C_30_H_46_O_4_	14.010	471.3535	453.3387, 435.3291, 399.3260, 381.2784	ZX	Chang et al. (2021)
8	33.02	(E)-2-(3,7-dimethylocta-2,6-dien-1-yl)-4-methoxy-6-methylphenol	C_18_H_26_O_2_	1.030	275.2008	231.1384, 205.1229, 177.0916, 151.0752	BZ	Zhang et al. (2018)
9	33.40	3*β*-acetoxy atractylon	C_17_H_22_O_3_	1.268	275.1645	215.1432, 197.1326, 145.1016	BZ	Zhang et al. (2018)
10	33.16	16-oxo-alisol A	C_30_H_48_O_6_	0.662	505.3527	487.3388, 469.3321, 451.3203, 415.2840, 397.2732	ZX	Liu et al. (2010)
11	33.19	2-(4a-methyl-8-methylene1, 4,4a,56,7,8,8a-octahydronaphthalen-2-yl)-acrylic acid	C_15_H_20_O_2_	0.744	233.1539	215.1432, 187.1483, 177.0911, 159.1171, 145.1014	BZ	Chang et al. (2021)
12	33.68	Atractylenolide I	C_15_H_18_O_2_	2.309	231.1385	203.1432, 189.0911, 185.1329, 175.0756, 161.0600, 147.0810, 135.0441	BZ	Chang et al. (2021)
13	35.41	Carvenone	C_10_H_16_O	−2.548	153.1275	135.1173, 109.1014, 107.0857, 95.0858	ZX	Zhang et al. (2018)
14	36.63	Aliso F	C_30_H_48_O_5_	1.060	489.3583	471.3473.453.3371, 399.2897, 381.2788	ZX	Liu et al. (2010)
15	36.89	16-oxo-alisol A 24-acetate	C_32_H_50_O_7_	−4.148	547.3607	529.3503, 511.3419, 493.3301, 415.2998	ZX	Liu et al. (2010)
16	36.91	Alisol C 23-acetate	C_32_H_48_O_6_	0.011	529.3524	511.3402, 469.3300, 451.3216, 433.3097, 415.2985, 397.2874	ZX	Chang et al. (2021)
17	37.02	Alisol K/J	C_30_H_44_O_5_	9.476	485.3307	467.3148, 449.3040, 431.2938, 353.2473	ZX	Na Yang et al. (2020)
18	39.01	Alisol B	C_30_H_48_O_4_	−5.908	473.3597	455.3488, 437.3436, 383.2946, 339.2687	ZX	Liu et al. (2010)
19	39.61	Alisol B 23-acetate	C_32_H_50_O_5_	3.490	515.3749	497.3636, 479.2802, 437.3417, 419.3316, 383.2939	ZX	Liu et al. (2010)
20	41.14	7-[4-(11-hydroxy-undecyloxy)-phenyl]-7-pyridin-3-yl-hept-6-enoic acid ethyl ester	C_31_H_45_NO_4_	−3.516	496.3404	478.3295, 419.2558, 184.0737, 104.1071	BZ	Chang et al. (2021)
21	41.34	Oleic acid	C_18_H_34_O_2_	−1.121	281.2480	263.2374	BZ	Zhang et al. (2018)
22	42.90	22-hydroxy-alisol A	C_30_H_50_O_6_	−0.363	507.3684	471.3472, 453.3399, 435.3243, 417.3080, 399.2885, 381.2785, 339.2885	ZX	Li et al. (2019)
23	44.46	Alisol I	C_30_H_46_O_3_	−5.384	455.3495	437.3417, 419.3316, 383.2965, 365.2837, 341.2838, 339.2685	ZX	Na Yang et al. (2020)
24	44.73	Alisol A	C_30_H_50_O_5_	4.040	513.3571	497.3630, 473.3658, 455.3546, 437.3419, 383.2957	ZX	Liu et al. (2010)
25	47.49	Alisol L	C_30_H_44_O_4_	7.423	469.3347	451.3269	ZX	Liu et al. (2010)

Parts of representative and potential bio-active compounds in ZX and BZ were chosen to clarify their second-order mass spectrometry graphs and proposed fragmentation pathways, including 16-oxo-alisol A, alisol B, alisol B 23-acetate, alisol C 23-acetate, alisol F, alisol I, atractylenolide I, II and III (Liu et al., 2010; Chao et al., 2016; Chen et al., 2016; Li et al., 2016; Bi et al., 2017; Na Yang et al., 2020; Kanno et al., 2017; Fei et al., 2018; Zhang et al., 2018; Chang et al., 2021). Detailed descriptions were as follows.

#### 3.3.1 16-Oxo-alisol A

**FIGURE 3:**
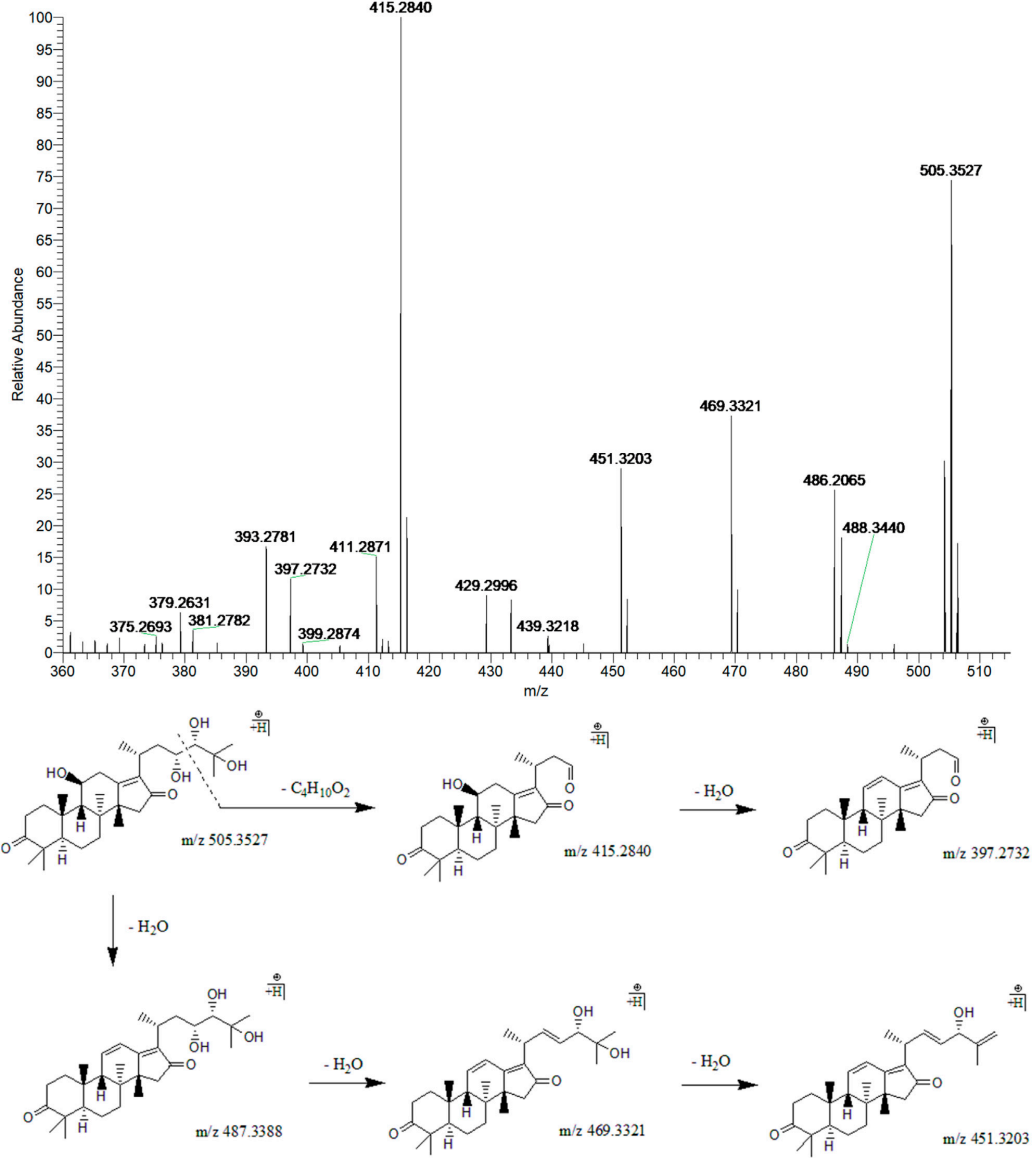
The second-order mass spectrometry graphs and proposed fragmentation pathways of 16-oxo-alisol A (peak 10).

Peak 10 (t_R_ = 33.16 min) displayed a quasi-molecular ion peak [(M + H)^+^] at m/z 505.3527 (C_30_H_49_O_6_)^+^, which was putatively determined to be 16-oxo-alisol A. Fragment ions at m/z 487.3388, 469.3321, and 451.3203 were ascribed to three dehydrations taking place at 11-OH, 23-OH, and 25-OH groups. Moreover, the fragment ion at 90 Da at m/z 415.2850 was presumably formed following the cleavage of the C23-C24 chemical bond from (C_30_H_49_O_6_)^+^, one of the characteristic fragmentations of alisol A derivatives, 16-oxo-alisol A included. Further dehydration occurred at 11-OH of the fragment ion at m/z 415.2850, which formed the fragment ion at m/z 397.2732. Thus, after comprehensive analysis, we putatively identified peak 10 as 16-oxo-alisol A (Figure 3).

#### 3.3.2 Alisol B and Alisol B 23-Acetate

**FIGURE 4:**
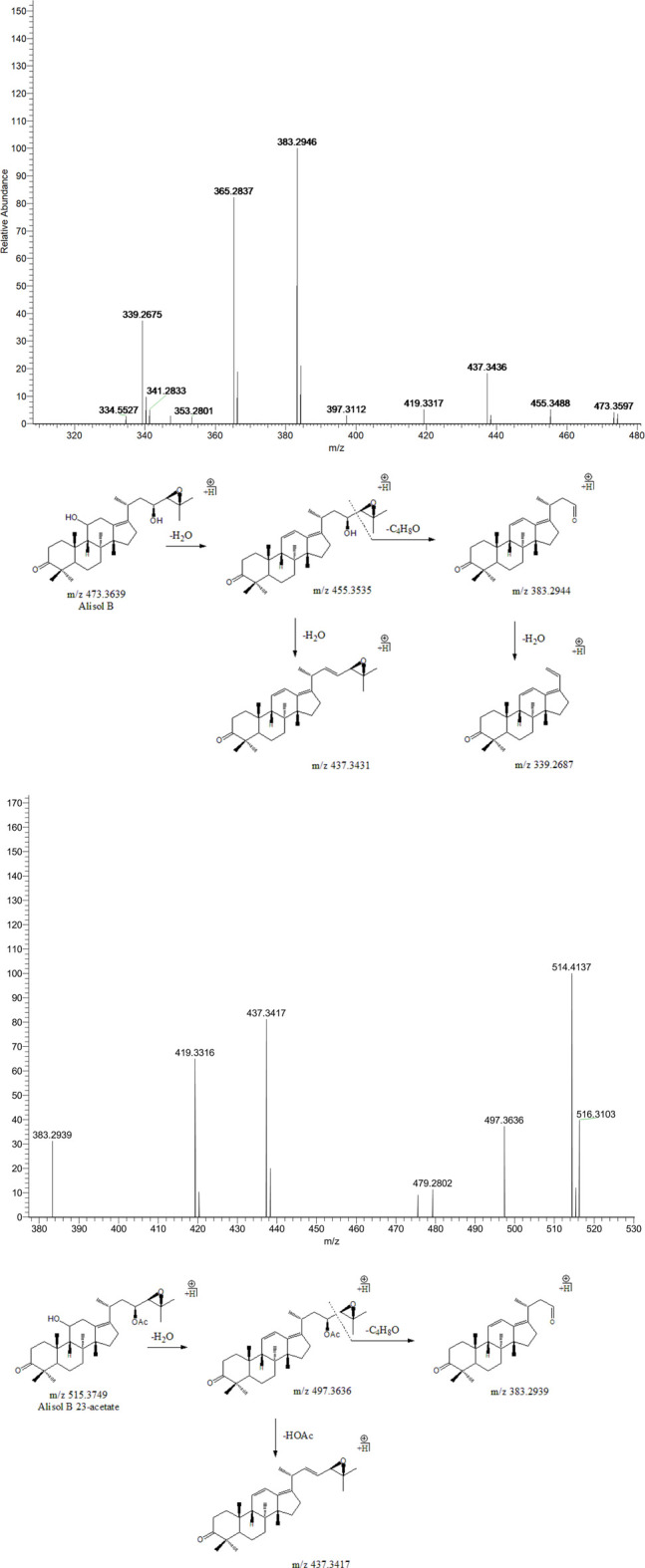
The second-order mass spectrometry graphs and proposed fragmentation pathways of alisol B and alisol B 23-acetate (peaks 18 and 19).

Peak 18 (t_R_ = 39.01 min) displayed a quasi-molecular ion peak [(M + H)^+^] at m/z 473.3597 (C_30_H_48_O_4_)^+^, which was putatively identified as alisol B. A series of dehydrations and the breaking of the C23-C24 chemical bond formed fragment ions at m/z 455.3488, 437.3436, and 383.2946, which was similar to the characteristic fragmentation process of 16-oxo-alisol A. Moreover, the fragment ion at m/z 339.2687 was attributed to the hydrogen-rearrangement in the ion at m/z 383.2946, and thus peak 18 was putatively identified as alisol B. Similarly, Peak 19 (t_R_ = 39.61 min) displayed a quasi-molecular ion peak [(M + H)^+^] at m/z 515.3749 (C_32_H_50_O_5_)^+^. Just as peak 18, fragment ions at m/z 497.3636 and 383.2939 were detected for dehydration from the parent ion and breaking of the C23-C24 chemical bond. Fragment ion at m/z 437.3417 could generate from that at m/z 497.3636 for deacetylation at 23-OAc. Thus, we putatively identified peak 19 as alisol B 23-acetate (Figure 4).

#### 3.3.3 Alisol C 23-Acetate

**FIGURE 5:**
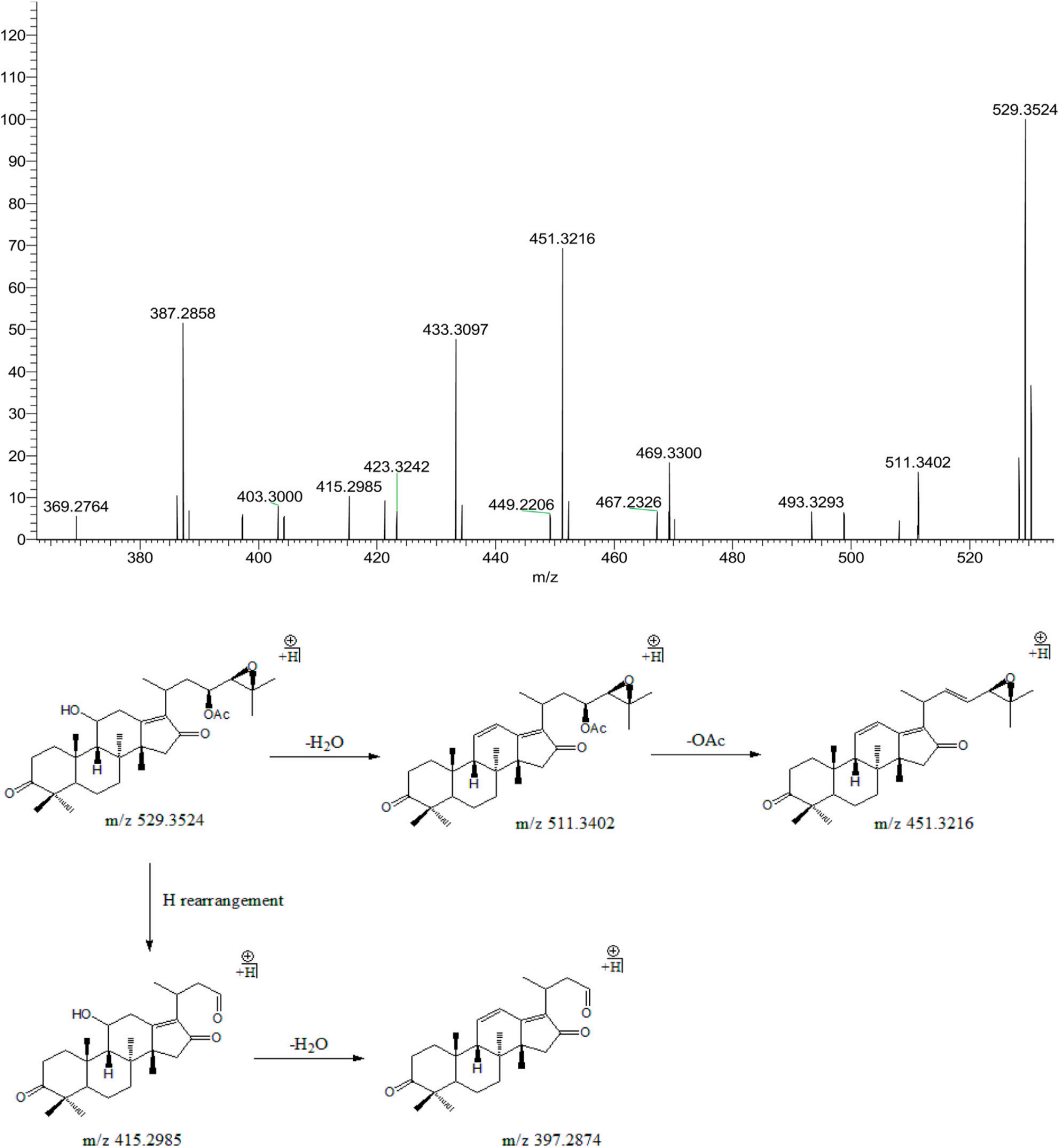
The second-order mass spectrometry graphs and proposed fragmentation pathways of alisol C 23-acetate (peak 16).

Peak 16 (t_R_ = 36.91 min) displayed a quasi-molecular ion peak [(M + H)^+^] at m/z 529.3524 (C_32_H_48_O_6_)^+^, which was putatively identified as alisol C 23-acetate. Fragment ion at m/z 511.3402 was putatively identified as arising from dehydrations at 11-OH. Similar to alisol B 23-acetate, fragment ion at m/z 451.3216 was detected for deacetylation at 23-OAc. Fragment ions at m/z 415.2985 and 397.2874 were formed because of further cleavage of C23-C24 chemical bond and dehydration from that at m/z 451.3216. Therefore, peak 16 was putatively identified as alisol C 23-acetate (Figure 5). The only difference between alisol C and alisol C 23-acetate was the presence of acetyl group. Thus, peak 5 that exhibited similar second-order mass characteristics as peak 16 was putatively identified as alisol C.

#### 3.3.4 Alisol F

**FIGURE 6:**
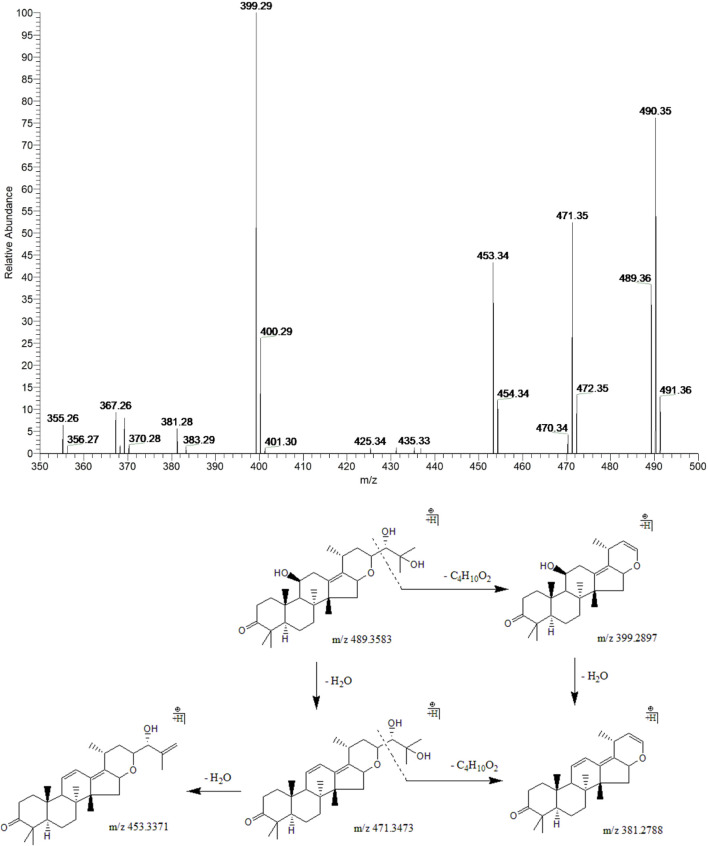
The second-order mass spectrometry graphs and proposed fragmentation pathways of alisol F (peak 14).

Peak 14 (t_R_ = 36.63 min) displayed a quasi-molecular ion peak [(M + H)^+^] at m/z 489.3583 (C_30_H_49_O_5_)^+^, which was putatively established as alisol F. Fragment ions at m/z 471.3473 and 453.3371 were detected for two dehydrations at 11-OH and 25-OH groups. Fragment ions at m/z 399.2897 and 381.2788 were formed for the breaking of the C23-C24 bond in the ions responsible for the peaks at m/z 489.3583 and 471.3473, respectively. In addition, fragment ion at m/z 381.2788 could also generate from dehydration at 11-OH of the ion at m/z 399.2897. Therefore, peak 14 was putatively identified as alisol F (Figure 6).

#### 3.3.5 Alisol I

**FIGURE 7:**
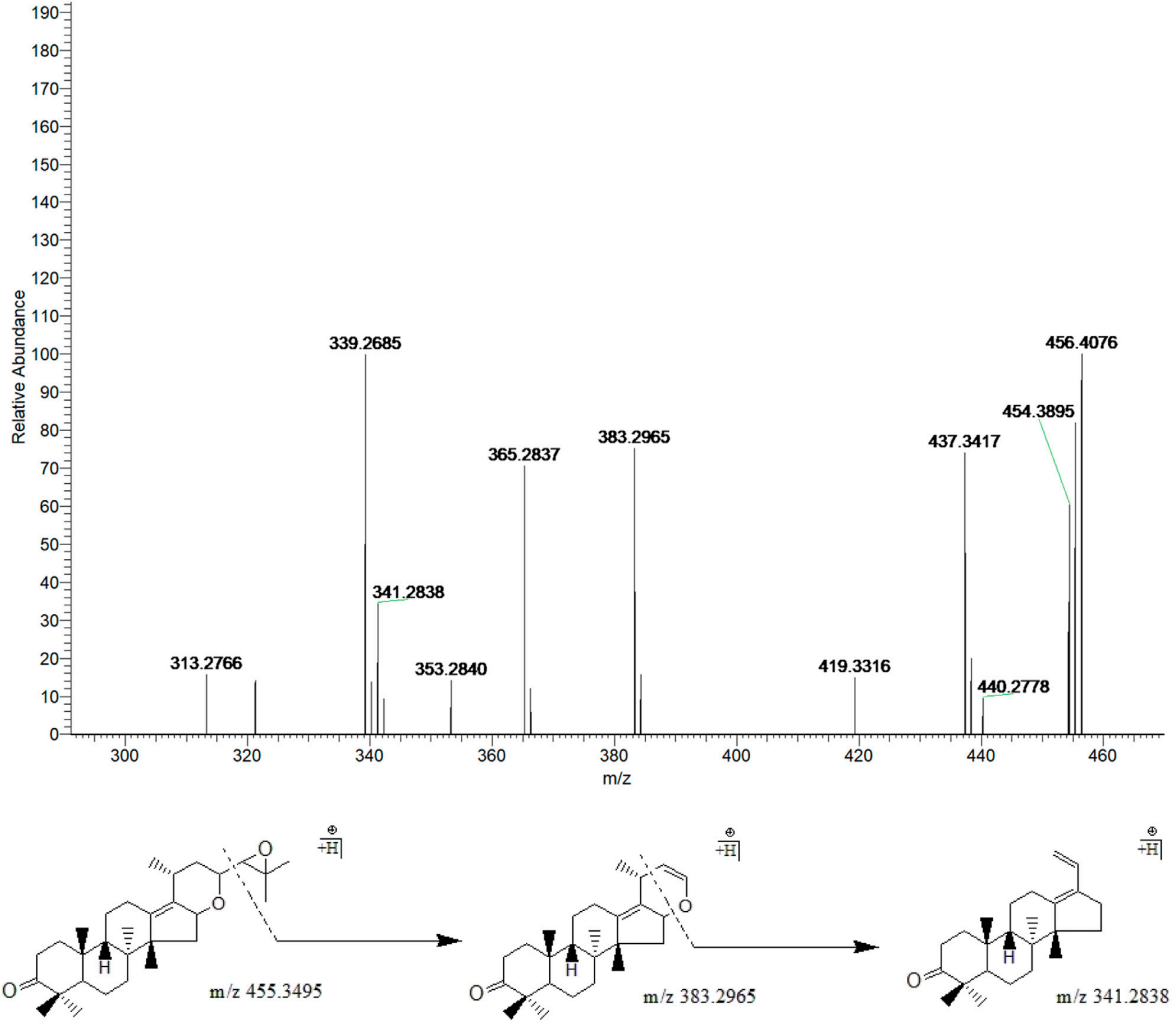
The second-order mass spectrometry graphs and proposed fragmentation pathways of alisol I (peak 23).

Peak 23 (t_R_ = 44.46 min) displayed a quasi-molecular ion peak [(M + H)^+^] at m/z 455.3495 (C_30_H_46_O_3_)^+^, which was deduced as alisol I. Fragment ion at m/z 383.2965 was putatively identified as arising from the cleavage of C23-C24 chemical bond, which was the same as alisol F. Fragment ion at m/z 341.2838 formed by the ion-source dissociation of the 16,23-oxide 6-membered ring of that at m/z 383.2965, and thus peak 23 was putatively identified as alisol I. (Figure 7).

#### 3.3.6 Atractylenolide I, II, and III

**FIGURE 8:**
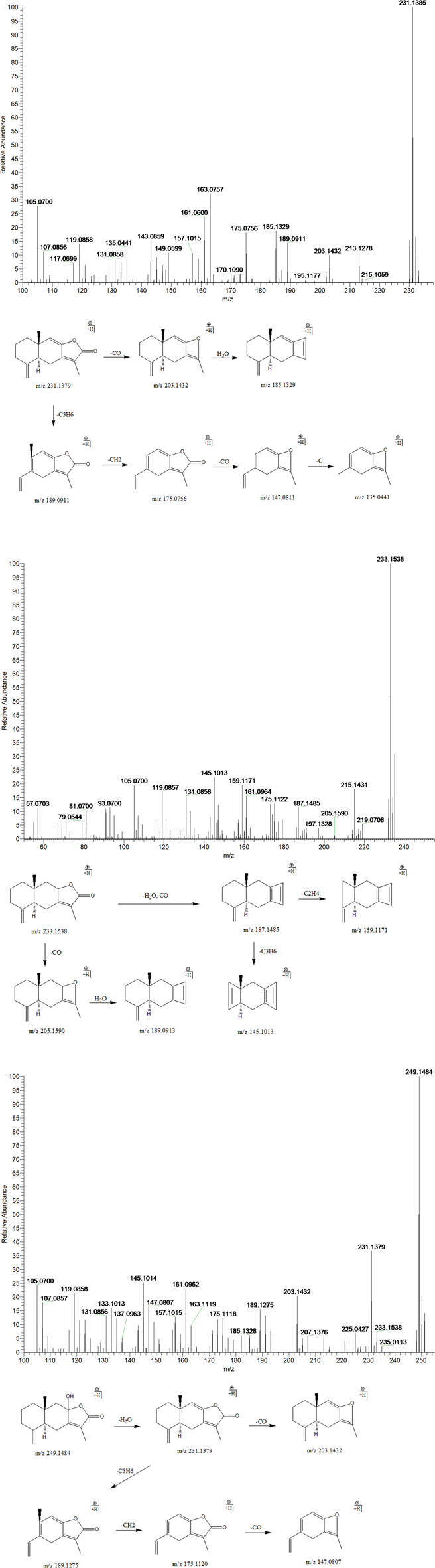
The second-order mass spectrometry graphs and proposed fragmentation pathways of atractylenolide I, II, and III (peaks 3, 4, and 12).

Peak 3, 4 and 12 (t_R_ = 24.21, 25.68 and 33.68 min) displayed quasi-molecular ion peaks at m/z 249.1484 (C_15_H_20_O_3_)^+^, 233.1538 (C_15_H_20_O_2_)^+^and 231.1385 (C_15_H_18_O_2_)^+^, which were putatively determined as atractylenolide III, II and I, respectively. Atractylenolide belongs to the family of sesquiterpene lactone compound, which existed ubiquitously in BZ. The existence of γ-lactone moieties can be authenticated by the characteristic fragment ions like (M + H-H_2_O)^+^ and (M + H-H_2_O-CO)^+^, which further cleave into characteristic fragment ions by the loss of CH_2_, C_2_H_4_, C_3_H_6,_ and C_4_H_8_ in the 12-membered ring of the parent ion. By screening the MS/MS spectrum of peak 3, fragment ions like (M + H-H_2_O)^+^, (M + H-H_2_O-CO)^+^, (M + H-H_2_O-C_3_H_6_)^+^, (M + H-H_2_O-C_4_H_8_)^+^, and (M + H-H_2_O-C_4_H_8_-CO)^+^ were generated at m/z 231.1379, 203.1432, 189.1275, 175.1120, and 147.0807, respectively. Similarly, peak 4 generated fragment ions like (M + H-H_2_O)^+^, (M + H-H_2_O-CO)^+^, (M + H-H_2_O-CO-C_2_H_4_)^+^, and (M + H-H_2_O-CO-C_3_H_6_)^+^ at m/z 215.1431, 187.1485, 159.1171, and 145.1013, respectively. Moreover, peak 12 exhibited comparable fragmentation patterns in line with peaks 3 and 4. Therefore, they were putatively identified as atractylenolide I, II and III, respectively (Figure 8).

### 3.4 Results of Quantitative Analysis

The quantitative analysis of alisol A, alisol A 24-acetate, and alisol B in the ethanol extract of ZXD was performed using validated UPLC-Q-TRAP-MS/MS methods. The three constituents’ contents were determined using their respective calibration curves. As a result, the alisol A, alisol A 24-acetate, and alisol B concentrations in ZXD were determined to be 1.5506, 0.4276, and 1.7571 mg/g, respectively (raw plant equivalent).

### 3.5 Results of the Pharmacokinetic Study

**FIGURE 9:**
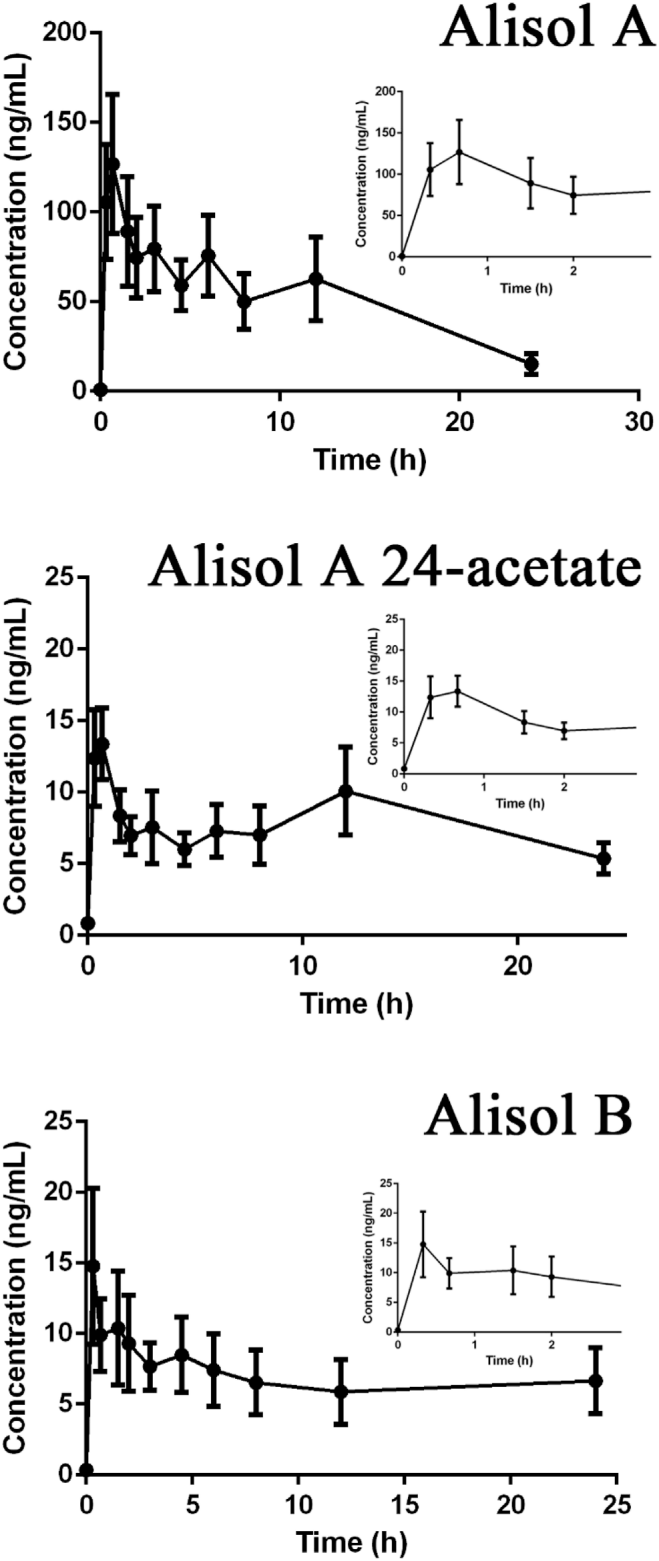
The pharmacokinetic profiles of alisol A, alisol A 24-acetate, and alisol B.

Drug-containing plasma samples collected in pre-dose and eleven different time points precisely; 20 min, 40 min, 1.5, 2, 3, 4.5, 6, 8, 10, 12, and 24 h were analyzed using UPLC-Q-TRAP-MS/MS to obtain the pharmacokinetic profiles of alisol A, alisol A 24-acetate and alisol B (Figure 9). GraphPad Prism software was used to visualize the mean plasma concentration-time curves. The time following administration was plotted on the X-axis, and the blood concentrations of the three constitutions were plotted on the Y-axis. The pharmacokinetic parameters were listed in Table 7. The results suggested that alisol A and alisol 24-acetate had comparable profiles with a slow-release impact 12 h after administration, which may be explained by their similar structures. All three constitutions were absorbed well orally in rats, and the analysis assay was sensitive and precise.

**TABLE 7:** The pharmacokinetic parameters of animals administered ZXD at a single dose of 750 mg/kg.

	T_max_ (h)	C_max_ (ng/ml)	C_max__D (kg*ng/ml/mg)	C_last_ (ng/ml)	AUC_last_ (h*ng/mL)	AUC_all_ (h*ng/mL)	AUMC_last_ (h*h*ng/mL)	MRT_last_ (h)
Alisol A	2.111 ± 1.968	160.733 ± 80.949	138.205 ± 69.603	18.958 ± 11.072	1,362.485 ± 697.656	1,384.785 ± 681.549	11,011.313 ± 6,355.099	8.036 ± 2.35
Alisol A 24-acetate	4.471 ± 5.337	30.893 ± 19.4	20.874 ± 17.056	7.928 ± 4.858	372.829 ± 298.265	372.829 ± 298.265	4,051.226 ± 3,161.536	11.152 ± 1.278
Alisol B	3.055 ± 4.116	16.876 ± 6.799	52.624 ± 21.202	5.361 ± 2.409	187.816 ± 72.501	187.816 ± 72.501	2077.655 ± 664.85	11.634 ± 1.856

## 4 Conclusion

In summary, rapid, sensitive, and accurate methods were successfully developed for qualitative and quantitative analysis of the ethanolic extract of ZXD. Twenty-five chemical constituents of ZXD were putatively identified in plasma using UHPLC-QE-Orbitrap-MS, three of them were further quantified and pharmacokinetically studied using UHPLC-Q-TRAP-MS/MS. Alisol A, alisol A 24-acetate, and alisol B were present in ZXD at concentrations of 1.5506, 0.4276, and 1.7571 mg/g (raw plant equivalent), respectively, and their pharmacokinetic profiles were presented. Our study will establish a theoretical base for future research and clinical applications of ZXD.

## Data Availability

The original contributions presented in the study are included in the article/Supplementary Material, further inquiries can be directed to the corresponding author.
