# Supplementary material for: Qualitative Analysis of Drug-Containing Plasma and its Application to Quantitative Analysis and Pharmacokinetic Study of Zexie Decoction Using UPLC-MS/MS
Source: Front Chem. 2022 Feb 22;10:815886. doi: 10.3389/fchem.2022.815886 (PMC8901485; doi:10.3389/fchem.2022.815886)
Supplement: Supplementary file 1 [file DataSheet1.docx]

**Figure S1.** The LOQ of alisol A

**Figure S2.** The LOQ of alisol B

**Figure S3.** The LOQ of alisol A 24-acetate

**Figure S4.** The LOD of alisol A

**Figure S5.** The LOD of alisol B

**Figure S6.** The LOD of alisol A 24-acetate


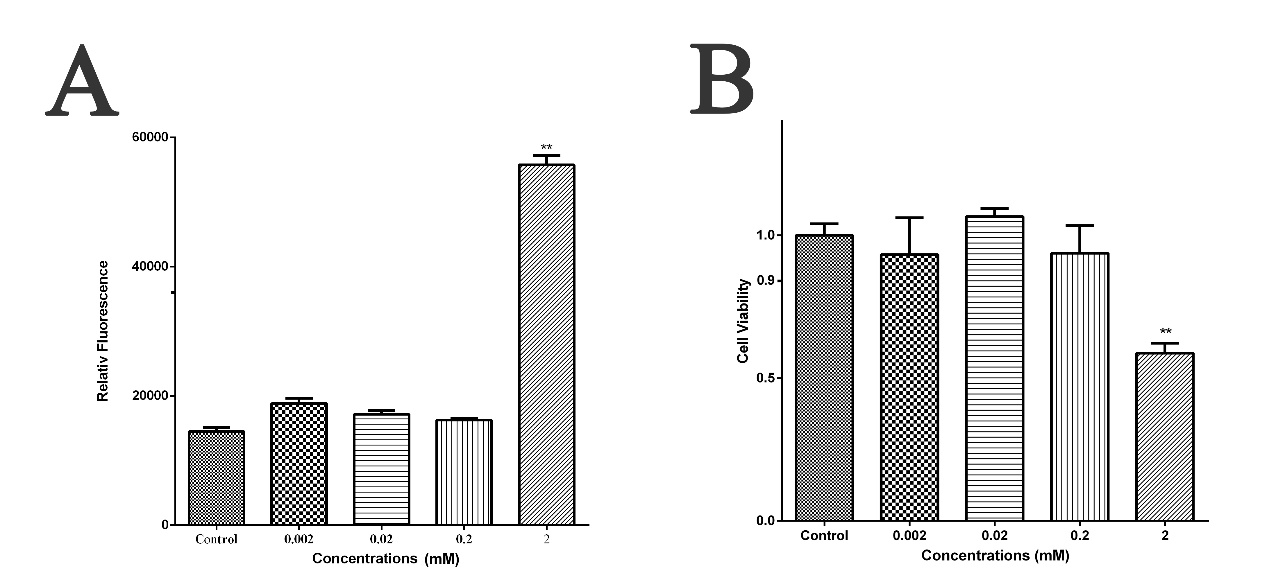


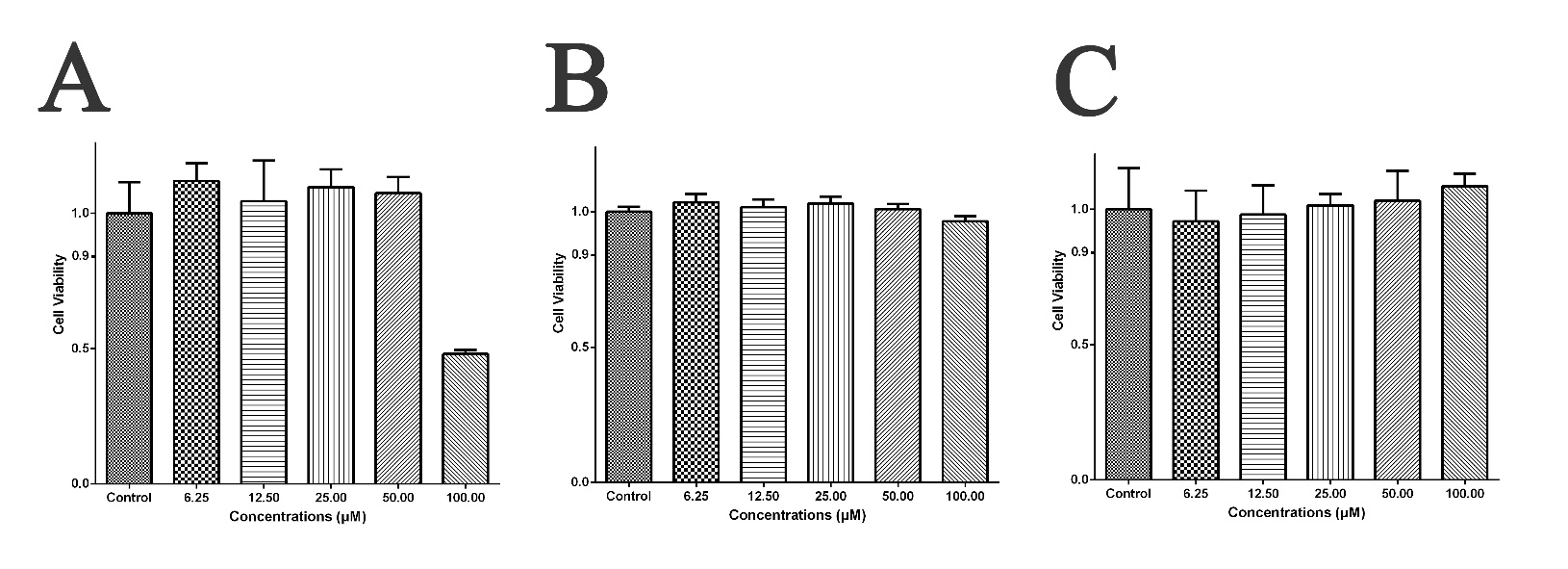
**Figure S7.** The lipid accumulation model induced by different concentrations of oleic acid in HepG_2_ cells. The optimal modeling concentration was 2 mM. A. The relative fluorescence of HepG_2_ cells administered oleic acid; B. The cell viability of HepG_2_ cells administered oleic acid. Control, HepG_2_ cells without treatment. **p<0.01, compared with the control group.


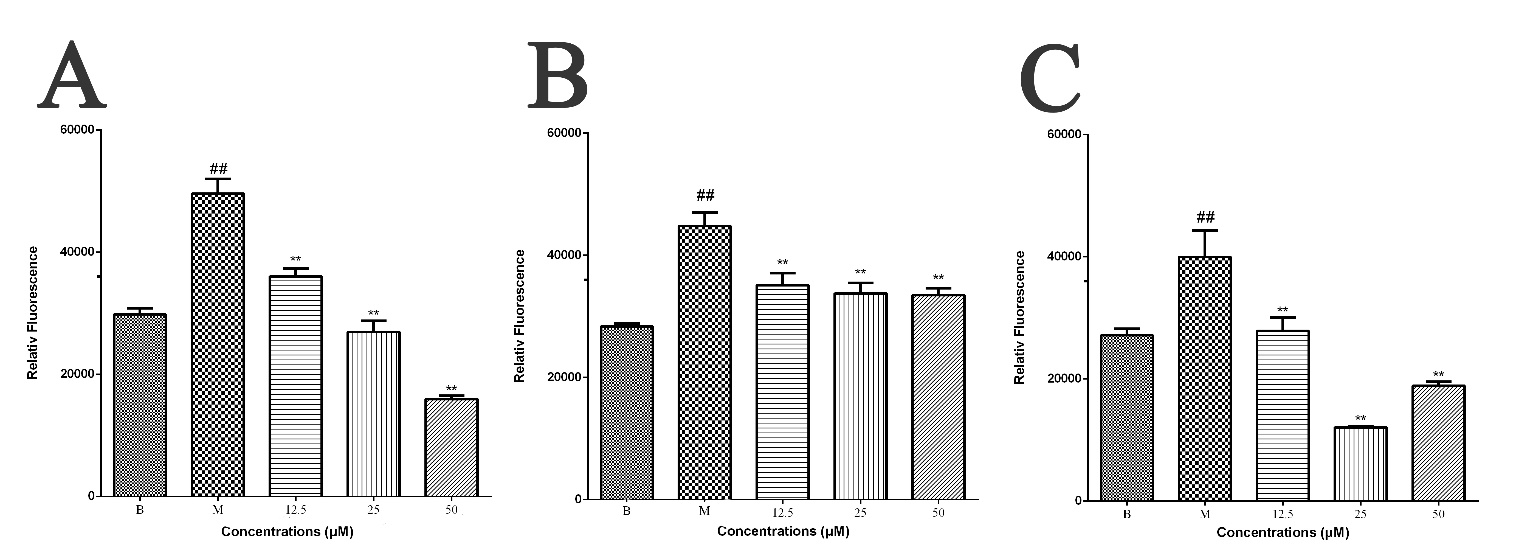
**Figure S8.** The cell viability of HepG_2_ cells administered alisol A (A), alisol A 24-acetate (B), and alisol B (C) at different concentrations. The cell viability was greater than 90% at a concentration range of 6.25-50.00. Control, HepG_2_ cells without treatment.

**Figure S9.** The lipid-lowing effect of alisol A (A), alisol A 24-acetate (B), and alisol B (C) in HepG_2_ cells. B, blank group, HepG_2_ cells without treatment; M, model group, HepG_2_ cells administered oleic acid at a concentration of 2mM; ^##^p<0.01, compared with the blank group; **p<0.01, compared with the model group.
